# Morphological Variability and Function of Labial Cartilages in Sharks (Chondrichthyes, Elasmobranchii)

**DOI:** 10.3390/biology12121486

**Published:** 2023-12-03

**Authors:** Claudia Klimpfinger, Jürgen Kriwet

**Affiliations:** Department of Paleontology, University of Vienna, 1090 Vienna, Austria; juergen.kriwet@univie.ac.at

**Keywords:** feeding mechanisms, feeding strategies, jaw morphology, suction, ram feeding, feeding behavior

## Abstract

**Simple Summary:**

Labial cartilages (LCs) are structures along the jaws of many sharks that are known, in some species, to enable or support suction, but there are many different shapes, numbers, and positions of LCs. This study is a morphological description of the large variety of LCs in sharks and an estimation of their suction potential according to the data of species known for their suction feeding. This additional information on their feeding strategies may help to accurately protect endangered shark species.

**Abstract:**

(1) Background: Labial cartilages (LCs), as their name suggests, lie in the folds of the connective tissue, the lips, framing the gape of elasmobranch chondrichthyans. As such, these cartilages lie laterally to the jaws and marginal teeth. They are considered to influence the ability of creating suction during the feeding process. As past studies have shown, LCs in sharks are as diverse as their varied feeding techniques and differ between species in number, size, shape, and position. This allows establishing parameters for inferring the feeding and hunting behaviors in these ecologically important fishes. (2) Methods: We present a study of LCs based on the CT scans of more than 100 extant shark species and, therefore, represent at least one member of every living family within the Euselachii, excluding batoids. (3) Results: Accordingly, sharks without labial cartilages or that have only small remnants are ram feeders or use pure biting and mainly occupy higher trophic levels (tertiary and quaternary consumers), whereas suction-feeding sharks have higher numbers (up to five pairs) of well-developed LCs and occupy slightly lower trophic levels (mainly secondary consumers). Species with unique feeding strategies, like the cookie-cutter shark (*Isistius brasiliensis*, an ectoparasite), display distinct shapes of LCs, while generalist species, conversely, exhibit a simpler arrangement of LCs. (4) Conclusions: We propose a dichotomous identification key to classify single LCs into different morphotypes and propose combinations of morphotypes that result in suction feeding differing in strength and, therefore, different hunting and feeding strategies. The conclusions of this study allow to infer information about feeding strategies not only in extant less-known sharks but also extinct sharks.

## 1. Introduction

**Scheme 1:**
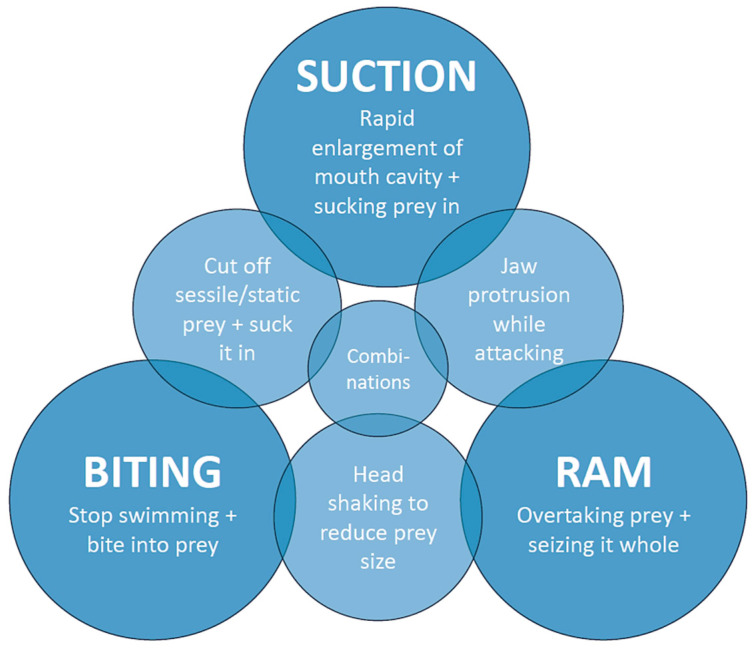
Main feeding mechanisms of sharks, including common intermediate forms and possible combinations.

The group Elasmobranchii (sensu [1]; Neoselachii sensu [2]) encompasses all modern sharks, rays, and skates in a monophyletic clade [1,2]. The fossil records of Elasmobranchii [1] extend back to the Early Permian period (ca. 295 mya) [3] and they adapted to a wide range of niches during their evolutionary history. This resulted in the development of various feeding mechanisms [4] (Scheme 1) as exemplified in apex predators, like the great white shark (*Carcharodon carcharias*) and the tiger shark (*Galeocerdo cuvier*); small, highly adapted species, like the cookie-cutter shark (*Isistius brasiliensis*) hunting in an ectoparasitic way; the Japanese wobbegong (*Orectolobus japonicas*) being a very sufficient benthic living suction feeder; or giant plankton feeders, like the whale shark (*Rhincodon typus*). These niche adaptations required different developments in tooth and jaw structures to solve the requirements for prey capture. In this paper, we focus on the labial cartilages (LCs) of sharks. The LCs are paired structures located on both sides of the head, laterally to the jaws. Their position along the jaws varies and therefore constrains the mouth-gape to a higher or lesser extent. LCs are considered a shared derived character as they are present in extinct shark species [5,6,7,8,9,10] and their sister groups [11,12,13,14], supporting that these structures are symplesiomorphic for living sharks. They vary in number (0–5 pairs), size and orientation, and have been described previously in some species, e.g., [8,15,16,17], but only insufficiently or not at all in others, e.g., [18,19]. Their origin and function have been discussed for over 100 years (e.g., [20,21,22,23,24,25]), but since 2001, it is well established that LCs support suction during feeding in sharks [8]. Still, not all LCs enable suction [26], but a certain number and size is necessary to form an efficient tunnel for generating a suction flow. The objective of this work is to provide precise descriptions of the number, position, structure, and orientation of LCs in different shark species. Previously, Klimpfinger and Kriwet [26] introduced a general identification system for LCs in sharks and arranged extant shark families according to their jaw and LC structure into eight morphotypes (groups A to H). The present study represents an extension of this work providing novel information about the morphology and variability of LCs in sharks and installing a dichotomous identification key for different morphotypes of LCs. Secondly, we discuss the combinations of LC morphotypes that account for different suction performances, deduced from physiological and biomechanical studies, e.g., [8,16,27,28]. This provides an additional insight into the feeding mechanisms of sharks.

## 2. Materials and Methods

We used published CT and micro-CT scans as well as pictures from published studies resulting in a dataset of 120 shark species, for which information about the presence/absence and nature of labial cartilages could be retrieved. Thirty-four species of the dataset lack labial cartilages and subsequently were excluded from the study, resulting in 86 species (Supplementary Table S1). The main sources for the descriptions of LCs in the remaining 86 species were based on X-ray-computed scans provided in www.figshare.com (accessed on 24 October 2023) [29] by Kamminga et al. [30] and the Chondrichthyan Tree of Life [31] by Corrigan, Naylor, Yang et al. [32]. Additionally, we retrieved information for the whale shark (*Rhincodon typus*) from Denison [15]; the Greenland shark (*Somniosus microcephalus*) from White [33]; the Port Jackson shark (*Heterodontus portusjacksoni*) from Summers [34]; the blackbelly lanternshark (*Etmopterus lucifer*) from Staggl [35]; for the white shark (*Carcharodon carcharias*), the shortfin mako (*Isurus oxyrinchus*), the longfin mako (*Isurus paucus*), the salmon shark (*Lamna ditropis*), and the smalltooth sand tigershark (*Odontaspis ferox*) from Shimada [19]; and the megamouth shark (*Megachasma pelagios*) from Seigel [36] and Shimada [19]. This resulted in the representation of at least one species of each of the currently known nine shark orders (Hexanchiformes, Squaliformes, Squatiniformes, Pristiophoriformes, Echinorhiniformes, Heterodontiformes, Orectolobiformes, Lamniformes, and Carcharhiniformes) and almost one species for each of the currently identified families.

### Morphological Descriptions

**Figure 1:**
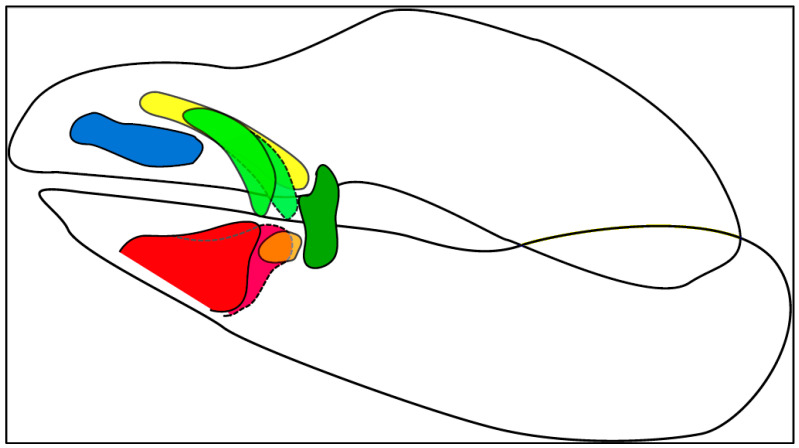
Hypothetical sketch of a shark jaw depicting all possible LCs in their resting positions. LC 1 = blue; LC 2 = green; LC 2.1 = yellow; LC 3.1 = orange; LC 3 = red.

**Figure 2:**
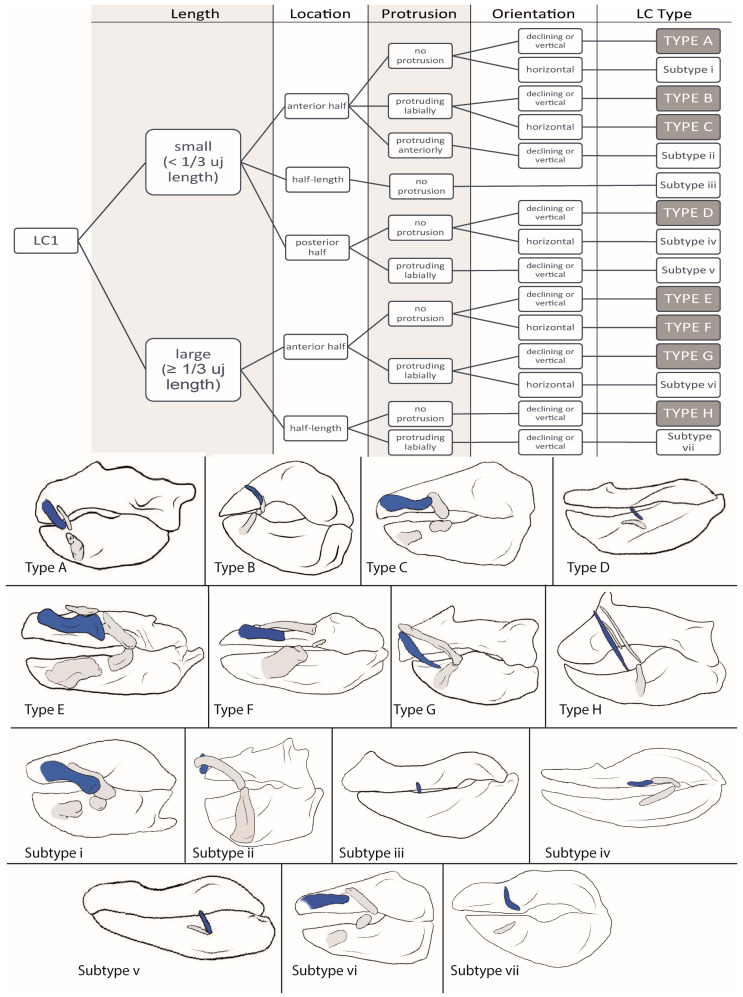
Dichotomous identification key for LC1 and sketches of example species of each LC type. Type A = *Oxynotus centrina*; Type B = *Stegostoma fasciatum*; Type C = *Hemiscyllium strahani*; Type D = *Negaprion brevirostris*; Type E = *Orectolobus japonicas*; Type F = *Squatina africana*; Type G = *Deania calcea*; Type H = *Scymnodalatias albicauda*; Subtype i = *Nebrius ferrugineus*; Subtype ii = *Dalatias licha*; Subtype iii = *Prionace glauca*; Subtype iv = *Chlamydoselachus anguineus*; Subtype v = *Mitsukurina owstoni*; Subtype vi = *Hemiscyllium trispeculare*; and Subtype vii = *Heterodontus francisci.*

**Figure 3:**
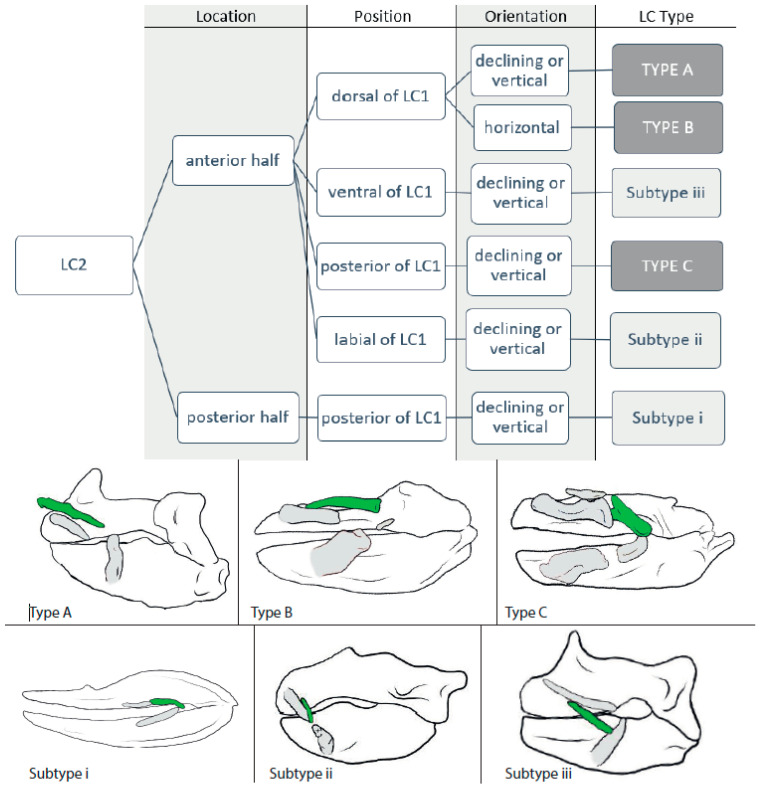
Dichotomous identification key for LC2 and sketches of example species of each LC type. Type A = *Squalus acanthias*; Type B = *Squatina africana*; Type C = *Orectolobus japonicas*; Subtype i = *Chlamydoselachus anguineus*; Subtype ii = *Oxynotus centrina*; and Subtype iii = *Squalus mitsukurii.*

**Figure 4:**
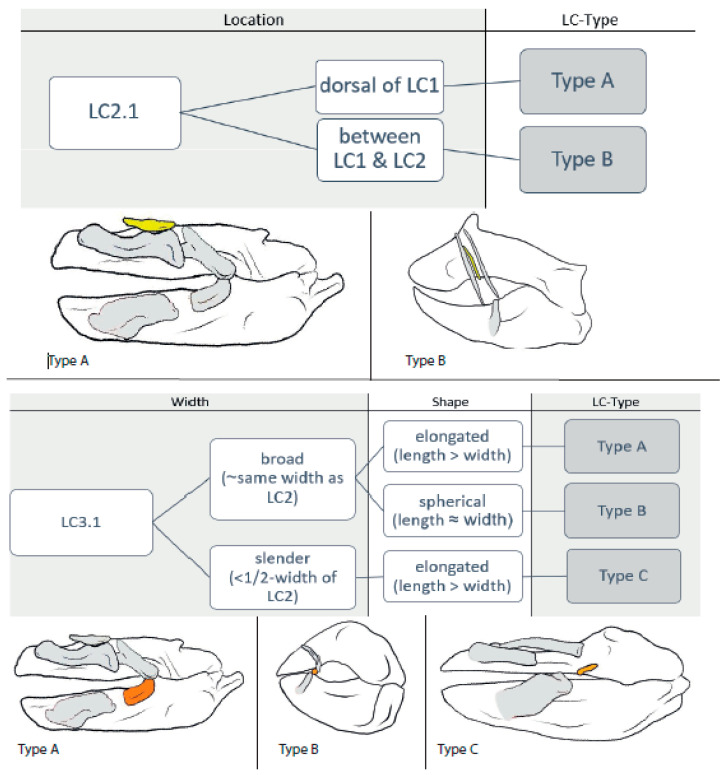
Dichotomous identification key for LC2.1 and LC3.1 and sketches of example species of each LC type. LC2.1: Type A = *Orectolobus japonicas*; and Type B = *Scymnodalatias albicauda*. LC3.1: Type A = *Orectolobus japonicas*; Type B = *Stegostoma fasciatum*; and Type C = *Squatina africana.*

**Figure 5:**
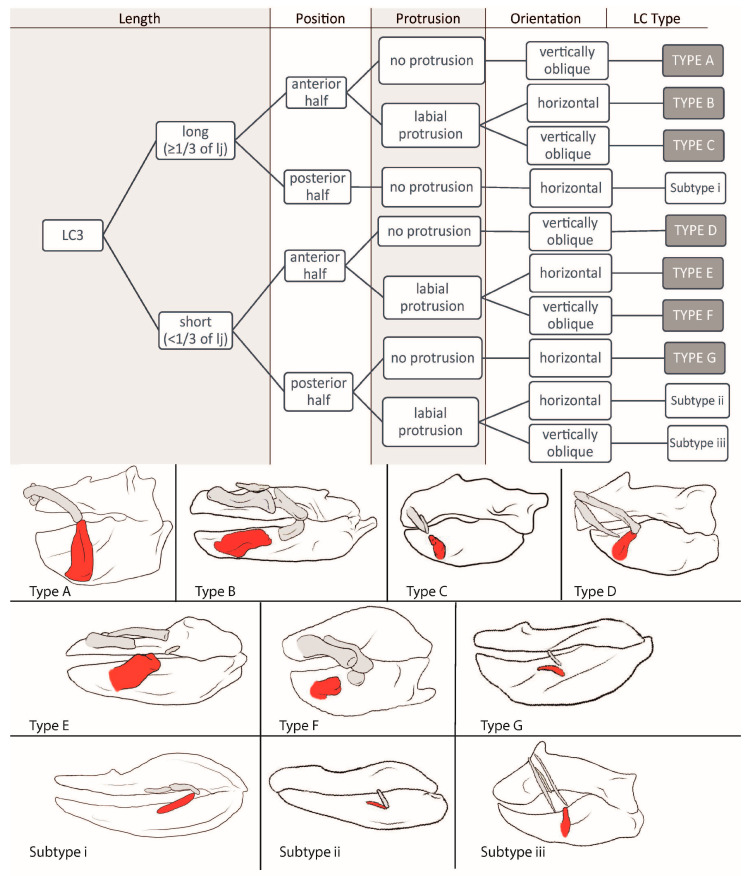
Dichotomous identification key for LC3 and sketches of example species of each LC type. Type A = *Dalatias licha*; Type B = *Orectolobus japonicas*; Type C = *Oxynotus centrina*; Type D = *Deania calcea*; Type E = *Squatina africana*; Type F = *Nebrius ferrugineus*; Type G = *Negaprion brevirostris*; Subtype i = *Chlamydoselachus anguineus*; Subtype ii = *Mitsukurina owstoni*; and Subtype iii = *Scymnodalatias albicauda.*

We downloaded 52 CT stacks from figshare.com and used the Amira software (https://www.thermofisher.com/at/en/home/electron-microscopy/products/software-em-3d-vis/amira-software.html, accessed on 24 October 2023) packages to reconstruct the scanned individuals and to mark the jaws and detectable LCs, which was also conducted for *Etmopterus lucifer*, of which a micro-CT scan was provided by Staggl [35]. We used the volume-rendering function for an initial overview; then, the jaws and LCs were marked using the orthogonal view and the brush tool, in order to visualize the structures. We rechecked the selected areas in the volume-rendering view and applied the surface-rendering tool to search for any LC we might have missed. We transferred the processed scans to the Autodesk program Sketchbook and used a stylus pen to highlight the LCs according to the assigned colors (see Table 1). The same was performed with the micro-CT-scan of *E. lucifer*. After reconstructing the jaws and LCs, we also compared our reconstructions to other reconstructions published in sharksrays.org to check for similarities and aberrations and added those species for which we did not have scans (29 species). For LC identifications, we employed the definitions of LCs of Klimpfinger and Kriwet [26], who numbered LCs as 1, 2, 2.1, 3, and 3.1 (Table 1; Figure 1). We also established a dichotomous identification key for the different morphotypes of LCs (Figure 2, Figure 3, Figure 4 and Figure 5).

The following descriptions always refer to the LC in the corresponding sub-heading and the position always refers to the onset of the LC or its anterior end. The total number of LCs for each species was added in brackets next to the species name. Types comprise three or more species; Subtypes contain a maximum of two species, except for LCs 2.1 and 3.1. Since only few species possess those two, all groups were considered types, independent of the species number included. The orientation of the LCs was described with “posteriorly declining” if the anterior end was positioned more dorsally than the posterior end (for example, see Figure 1 for LC 2.1). If the anterior end was positioned more ventrally than the posterior end, the orientation was described as “posteriorly inclining” (for example, see Figure 1 for LC 3).

## 3. Results

### 3.1. Morphological Descriptions

#### 3.1.1. LC1 (Figure 2)

**Type A:** The LCs1 of *Hexanchus nakamurai* (1), *Poroderma africanum* (1), *Alopias vulpinus* (1), *Scyliorhius canicula* (1), *Scyliorhinus meadi* (1), *Scyliorhinus boa* (1), *Scyliorhinus stellaris* (1), *Mustelus higmani* (1), *Mustelus asterias* (2), Atelomycterus macleayi (2), *Oxynotus centrina* (3), *Centrophorus seychellorum* (3), *Mollisquama parini* (3), *Isistius brasiliensis* (3), *Euprotomicrus bispinatus* (3), *Squalus acanthias* (3), and *Squatina nebulosa* (3) are small (<1/3 length of the upper jaw), located in the anterior half of the upper jaw in a posteriorly declining or vertical position, and do not protrude from the jaws. In *A. macleayi* and *H. nakamurai*, they display a convex bend, while in *P. africanum*, *C. seychellorum*, *S. nebulosa*, *S. canicula*, and *S. meadi*, they display a concave bend. In all other group members, there is no detectable bend. A ligamentous connection to the upper jaw is possible but not necessarily present.

**Type B:** In *Schroederichthys chilensis* (2), *Brachaelurus waddi* (3), *Chiloscyllium arabicum* (3), *Rhincodon typus* (3), *Squalus brevirostris* (3), *Squalus megalops* (3), *Stegostoma fasciatum* (4), and *Parascyllium collare* (4), the LCs1 are small, positioned in the anterior half of the upper jaw in a posteriorly declining orientation, and protrudes labially. Except for *R. typus*, the LCs1 of all members are slender (<½ length of the upper jaw) and they are tightly connected to the upper jaw in *B. waddi*, *S. megalops*, and *C. arabicum* by ligaments.

**Type C:** The hemiscyliid species *Chiloscyllium indicum* (3), *Chiloscyllium punctatum* (4), *Chiloscyllium griseum* (4), *Hemiscyllium occellatum* (4), and *Hemiscyllium strahani* (4) possess small LCs1 that are located in the anterior half of the upper jaw, oriented horizontally, and protrude labially.

**Type D:** The LCs1 of *Scoliodon laticaudus* (1), *Sphyrna lewini* (1), *Sphyrna zygaena* (1), *Carcharhinus galapagensis* (2), and *Negaprion brevirostris* (2) are small and slender, located in the posterior half of the upper jaw in a vertical or posteriorly declining position, and do not protrude from the jaw.

**Type E:** In *Triakis semifasciata* (2), *Galeorhinus galeus* (2), *Atelomycterus marmoratus* (2), *Mustelus manazo* (2) and *Mustelus mustelus* (2), *Centroscymnus crepidater* (3), *Centroscymnus owstonii* (3), *Scymnodon ringens* (3), *Centrophorus uyato* (3) and *Centrophorus tessellatus* (3), *Zameus squamulosus* (3), *Echinorhinus brucus* (3), *Squatina squatina* (3), and *Orectolobus japonicus* (5), the LCs1 are large (≥1/3 length of the upper jaw), located in the anterior half of the upper jaw in a posteriorly declining or vertical position, and without any protrusion. Only S. squatina displays a notch at the posterior end and, except for *S. ringens* and *G. galeus*, none of the LCs1 are connected to the upper jaw. In *C. crepidater*, *C. uyato*, and *T. semifasciata*, a concave bend is detectable; in *O. japonicas*, multiple bends (S-shaped) are detectable; all other species of this type do not display any bends in their LCs1.

**Type F:** *Galeus melastomus* (2), *Galeus sauteri* (3), *Ginglymostoma cirratum* (3), *Squatina africana* (4), *Eucrossorhinus dasypogon* (5), and *Orectolobus maculates* (5) possess large LCs1, located in the anterior half of the jaw in a horizontal position, without any labial or anterior protrusion. In *E. dasypogon*, *O. maculates*, and *G. cirratum*, multiple bends are detectable.

**Type G:** In *Apristurus macrostomus* (2), *Apristurus laurussonii* (2), *Etmopterus lucifer* (2), *Etmopterus sheikoi* (2), and *Etmopterus splendidus* (2), *Squalus suckleyi* (3), *Squalus mitsukurii* (3), *Squalus cubensis* (3), *Chiloscyllium hasselti* (3), and *Deania calcea* (3), the LCs1 are large, located in the anterior half of the upper jaw in a vertical or posteriorly declining position, and protrude labially. They all are slender and, in *S. suckleyi*, *C. hasselti*, *A. macrostomus*, and *A. laurussonii*, they are tightly connected to the upper jaw by ligaments. A convex bend is detectable in the LCs1 of *S. suckleyi* and *E. lucifer*, while in those of *A. macrostomus, S. mitsukurii*, and *D. calcea*, a concave bend is detectable. The others display no bends.

**Type H:** The LCs1 of *Carcharhinus macloti* (1), *Hemigaleus microstoma* (1), *Bythaelurus canescens* (2), *Odontaspis ferox* (2), *Chaenogaleus macrostoma* (2), *Hemipristis elongata* (2), *Leptocharias smithii* (3), and *Scymondalatias albicauda* (4) are large, located at half-length of the upper jaw in a posteriorly declining position, and without any protrusion. In *L. smithii* and *C. macloti*, they display a slight concave bend and are connected tightly to the upper jaw by ligaments, as is also the case in *H. microstoma*.

**Subtype i:** In *Nebrius ferrugineus* (3) and *Squatina japonica* (3), the LCs1 are small, located in the anterior half of the upper jaw in a horizontal position without protrusion. They are broad (≥½ length of the upper jaw) and display a concave bend.

**Subtype ii:** In *Dalatias licha* (3), the LCs1 are small, located in the anterior half in a posteriorly declining or vertical position, and protrude anteriorly.

**Subtype iii:** In *Prionace glauca* (1), *Pristiophorus nudipinnis* (1), and *Carcharhinus falciformis* (2), the LCs1 are small, slender (<½ length of the upper jaw), and without protrusion. In *P. glauca* and *C. falciformis*, they are oriented in a posteriorly declining position, while in *P. nudipinnis*, the LCs1 are oriented horizontally.

**Subtype iv:** The LCs1 of *Chlamydoselachus anguineus* (3) are small, slender, located in the posterior half of the lower jaw in a horizontal position, and display a concave bend.

**Subtype v:** *Mitsukurina owstoni* (2) has small, slender LCs1, located in the posterior half of the upper jaw in a vertical position and that protrude labially.

**Subtype vi:** In *Chiloscyllium plagiosum* (3) and *Hemiscyllium trispeculare* (4), the LCs1 are large (≥1/3 length of the upper jaw) and broad, located in the anterior half of the upper jaw in a horizontal position and protrude labially. They are tightly connected to the upper jaw.

**Subtype vii:** In *Heterodontus francisci* (2) and *Heterodontus japonicus* (2), the LCs1 are large, slender, located at half-length of the upper jaw in an almost vertical position, and protrude labially.

#### 3.1.2. LC2 (Figure 3)

**Type A:** In *Squalus cubensis* (3), *Squalus brevirostris* (3), *Squalus acanthias* (3), *Squalus megalops* (3), *Squalus suckleyi* (3), *Mollisquama parini* (3), *Leptocharias smithii* (3), the centrophorids *Centrophorus seychellorum* (3), *Centrophorus tessellatus* (3), *Centrophorus uyato* (3) and *Deania calcea* (3), the somniosids *Centroscymnus crepidater* (3), *Centroscymnus owstonii* (3), *Scymnodon ringens* (3), *Zameus squamulosum* (3), *Stegostoma fasciatum* (4), and *Scymnodalatias albicauda* (4) as well as in *Parascyllium collare* (4) and the ginglymostomatids *Ginglymostoma cirratum* (4), and *Nebrius ferrugineus* (4), the LCs2 are located in the anterior half of the upper jaw, dorsally to LC1, and in a vertical or posteriorly declining orientation. They are slender (<½ length of upper jaw), display a notch at the posterior end in the ginglymostomatids (*G. cirratum* and *N. ferrugineus*), *C. tessellatus*, and *S. brevirostris*, and is attached to the upper jaw in the somniosids *C. owstonii* and *C. crepidater* by ligaments.

**Type B:** The LCs2 in *Isistius brasiliensis* (3), *Dalatias licha* (3), and *Euprotomicrus bispinatus* (3) and the squatinids *Squatina nebulosa* (3), *Squatina squatina* (3), *Squatina japonica* (3), and *Squatina africana* (4) are located in the anterior tip of the upper jaw, with the onset dorsally to LC1 and extending posteriorly beyond the LC1. They are oriented horizontally, display no kinds of bends, and are, except for those of *S. nebulosa*, considered as broad (≥½ length of the upper jaw).

**Type C:** In *Brachaelurus waddi* (3), *Rhincodon typus* (3), the hemiscyliids *Chiloscyllium hasselti* (3), *Chiloscyllium plagiosum* (3), *Chiloscyllium indicum* (3), *Chiloscyllium arabicum* (3), *Chiloscyllium griseum* (4), *Chiloscyllium punctatum* (4), *Hemiscyllium strahani* (4), *Hemiscyllium trispeculare* (4), and *Hemiscyllium occellatum* (4) as well as in the orectolobid species *Eucrossorhinus dasypogon* (5), *Orectolobus japonicas* (5), and *Orectolobus maculates* (5), the LCs2 are located in the anterior half of the upper jaw, posteriorly to LC1, and oriented vertically or in a posteriorly declining position. The LCs2 are slender (<½ length of upper jaw), except for the orectolobids, in which they are broad and display either no or a slight convex bend. *Rhincondon typus* displays a notch and the orectolobid species display a forking at the posterior end.

**Subtype i:** In *Galeus sauteri* (3) and *Chlamydoselachus anguineus* (3), the LCs2 are located in the posterior half of the upper jaw, posteriorly to LC1. They are slender and oriented obliquely.

**Subtype ii:** *Echinorhinus brucus* (3) and *Oxynotus centrina* (3) have their LCs2 located in the anterior half of the upper jaw, laterally to the LCs1, and in a posteriorly declining orientation. The LCs2 are slender and without bends or notches.

**Subtype iii:** *Squalus mitsukurii* (3) possesses slender LCs2, located in the anterior half of the jaw, ventrally to LC1, and in a posteriorly declining orientation.

#### 3.1.3. LC2.1 (Figure 4)

**Type A:** In the orectolobid species *Eucrossorhinus dasypogon* (5), *Orectolobus japonicus* (5), and *Orectolobus maculates* (5), the LC2.1 are located dorsally to LC1.

**Type B:** In *Scymnodalatias albicauda* (4), the LC2.1 are located between LC1 and LC2 and are oriented almost vertically, as are LC1 and LC2.

#### 3.1.4. LC3.1 (Figure 4)

**Type A:** In *Chiloscyllum griseum* (4), *Chiloschyllum punctatum* (4), *Chiloscyllium plagiosum* (4), *Hemiscyllium occellatum* (4), *Hemiscyllium strahani* (4), and *Hemiscyllium trispeculare* (4) as well as in the orectolobids *Eucrossorhinus dasypogon* (5), *Orectolobus japonicus* (5), and *Orectolobus maculates* (5), the width of the LC3.1s is about the same as that of the LCs2 and, therefore, considered as ‘stout’. They are elongated (length > width), oriented posteriorly inclining, and make contact with LC2.

**Type B:** Among *Stegostoma fasciatum* (4) and the members of the ginglymostomatids, *Ginglymostoma cirratum* (4) and *Nebrius ferrugineus* (4), the LC3.1s are also stout, but comparably spherical (length ≈ width).

**Type C:** In *Squatina africana* (4), the LCs3.1 are slender (<½ width of LC2) and elongated (length > width).

#### 3.1.5. LC3 (Figure 5)

**Type A:** The LCs3 in *Etmopterus splendidus* (2), *Zameus squamulosus* (3), *Mollisquama parini* (3), *Dalatias licha* (3), *Isistius brasiliensis* (3), *Euprotomicrus bispinatus* (3), *Centroscymnus owstonii* (3), *Centroscymnus crepidater* (3), and *Centrophorus tessellatus* (3) are long (≥1/3 of the lower jaw length) and located in the anterior half of the jaw. They do not protrude from the jaw and are oriented vertically or obliquely either being directed anteriorly or posteriorly. No notch can be detected, and they are always tightly connected to the lower jaw by ligaments.

**Type B:** In *Atelomycterus macleayi* (2), *Atelomycterus marmoratus* (2), *Galeus melastomus* (2), *Galeus sauteri* (2), and *Schroederichthys chilensis* (2), and the orectolobids *Eucrossorhinus dasypogon* (5), *Orectolobus japonicas* (5), and *Orectolobus maculates* (5), the LCs3, again, are long and located in the anterior half of the jaw, but do protrude labially, are not connected to the lower jaw, and are oriented horizontally. Additionally, in orectolobid species, the LCs3 display a distinct notch or forking at their posterior end.

**Type C:** The LCs3 are long, located in the anterior half of the jaw, protrude labially, and are oriented vertically and obliquely either being directed anteriorly or posteriorly in *Etmopterus lucifer* (2), *Etmopterus sheikoi* (2), *Etmopterus splendidus* (2), *Oxynotus centrina* (3), *Squalus acanthias* (3), *Squalus megalops* (3), *Squalus brevirostris* (3), *Squalus cubensis* (3), *Squalus suckleyi* (3), *Squalus mitsukurii* (3), *Stegostoma fasciatum* (4), and *Parascyllium collare* (4).

**Type D:** Only in *Deania calcea* (3), *Centrophorus scychellorum* (3), and *Centrophorus uyato* (3) are the LCs3 are short (<1/3 of the lower jaw length) and do not protrude from, but are tightly connected to, the lower jaw. They are located in the anterior half of the jaw and oriented posteriorly inclining.

**Type E:** The LCs3 in *Galeorhinus galeus* (2), *Mustelus asterias* (2), *Mustelus mustelus* (2), *Mustelus higmani* (2), *Mustelus manazo* (2), *Odontaspis ferox* (2), *Hemipristis elongata* (2), *Apristurus laurussonii* (2), *Bythaelurus canescens* (2), *Leptocharias smithii* (3), *Brachaelurus waddi* (3), *Rhincodon typus* (3), *Squatina nebulosa* (3), *Squatina squatina* (3), *Squatina japonica* (3) *Squatina africana* (4), *Chiloscyllium plagiosum* (3), *Chiloscyllium hasselti* (3), *Chiloscyllium griseum* (4), *Chiloscyllium punctatum* (4), *Hemiscyllium trispreculare* (4), *Hemiscyllium strahani* (4), and *Hemiscyllium occellatum* (4) are short, located in the anterior half of the lower jaw, oriented horizontally, and protrude labially. They are roundish in cross-section, except for the LCs of the Squatina species, which are flat. Also, in all species of Squatina, Chiloscyllium, and Hemiscyllium, a notch at the posterior end is detectable.

**Type F:** In *Heterodontus francisci* (2), *Heterodontus japonicas* (2), *Apristurus macrostomus* (2), *Triakis semifasciata* (2), *Chiloscyllium indicum* (3), *Chiloscyllium arabicum* (3), *Ginglymostoma cirratum* (3), *Nebrius ferrugineus* (3), *Scymnodon ringens* (3), *Squatina japonica* (3), and *Echinorhinus brucus* (3), the LCs3 are short, located in the anterior half of the jaw, oriented vertically and obliquely either being directed anteriorly or posteriorly, and protrude labially from the jaw.

**Type G:** The carcharhinids *Carcharhinus falciformis* (2), *Carcharhinus galapagensis* (2), and *Negaprion bervirostris* (2) as well as the hemigaleid *Chaenogaleus macrosotma* (2) possess short LCs3 that are located in the posterior half of the jaw, do not protrude, and are oriented horizontally. They are not connected to the lower jaw, are flat, and do not display a notch at either end.

**Subtype i:** *Chlamydoselachus anguineus* (3) possesses a long (≥1/3 of lower jaw length) LCs3, which are located in the posterior half of the jaw, oriented horizontally, and are not connected to and do not protrude from the lower jaw.

**Subtype ii:** *Mitsukurina owstonii* (2) has short (<1/3 of the lower jaw length) LCs3, located in the posterior half, protrude labially, and are oriented horizontally, without any connection to the jaw or detectable notch.

**Subtype iii:** *Scymnodalatias albicauda* (4) has short LCs3 that are located in the posterior half of the jaw, protrude labially, are oriented posteriorly inclining, and are tightly connected to the lower jaw.

### 3.2. Interpretations

#### 3.2.1. LC1

LC **Types D** and **H** as well as **Subtypes iii** and **iv** are considered LC1 remnants and are not capable of supporting any kind of suction.

**Type A** and **Subtype i** correlate with a biting feeding behavior typical of scavengers or species feeding on immobile prey (as seen in *Oxynotus centrina* in [37]) if a maximum of two LCs is present; in cases with three LCs, a combination of biting and suction is suggested (as seen in *Scyliorhinus canicula* in [38]).

**Types B, C, E, F,** and **G** as well as **Subtypes ii** and **vi** are correlated with suction feeding of different strengths, and often occur in combination with another feeding technique (biting or ram) (as seen in *Etmopterus lucifer* in [35]). In Type F and Subtype vi, the suction is considered to be quite strong (as seen in *Chiloscyllium plagiosum* in [39]).

**Subtype v** only occurs in the goblin shark (*Mitsukurina owstoni*), which uses an extraordinary strong jaw protrusion for hunting, and Subtype vii only occurs in horn sharks (Heterodontidae) with their extraordinary massive labial tissues.

#### 3.2.2. LC2

**Types A** and **B** are considered to reinforce LC1 (Type B more and Type A less) or serve as a pivoting point and, therefore, suggest a distinct usage in suction feeding (as seen in *Squatina californica* in [40] and in *Isistius plutodus* in [41]).

**Type C** is aligned with LC1 and, therefore, is considered to enlarge the mouth cavity, which suggests a certain amount of suction (as seen in *Rhincodon typus* in [42]).

**Subtype i** is considered to be a remnant of a formerly mouth-corner-enforcing LC2 (as seen in *Chlamydoselachus anguineus* in [43]), and Subtypes ii and iii suggest for minor suction (as seen in *Oxynotus centrina* in [37]).

#### 3.2.3. LC2.1

**Type A** is found in species that create strong suction forces and probably reinforces LC1 so that it can endure higher pressures (as seen in *Orectolobus ornatus* in [44]).

**Type B** is only found in the whitetail dogfish (*Scymnodalatias albicauda*) and, since this is to date the only species with this kind of arrangement, we are not sure whether it is used for creating suction.

#### 3.2.4. LC3.1

**Types A** and **B** are stout and more or less roundish in cross-section, both serving as additional elongations of LCs3 and as extra hinges for a more roundish mouth opening when extended (as seen in *Orectolobus maculatus* in [27]).

**Type C** is found only in one species of squatinids (*Squatina africana*), and we consider it to be the first hint of the development of an additional LC.

#### 3.2.5. LC3

**Type A** is considered an anchor point and, therefore, is correlated with the possibility of establishing a longer lasting suction in combination with a biting movement (as seen in *Dalatias licha* in [45]).

**Type B** suggests for a strong suction; since it is oriented horizontally, it is quite moveable and long and sometimes provides a notch for a better joint function (as seen in *Orectolobus maculatus* in [27]).

**Types C, D,** and **F** are correlated with creating a medium suction force used in combination with other feeding techniques (as seen in *Squalus acanthias* in [44]). It also might correlate with an elongated duration of suction if the LC is directed anteriorly (as seen in *Etmopterus lucifer* in [35]).

**Type E** indicates a medium suction force if three or more LCs are expressed (as seen in *Chiloscyllium plagiosum* in [46]), but only minor suction if a maximum of two LCs is present (as seen in *Odontaspis ferox* in [19]).

**Type G** and **Subtypes i, ii,** and **iii** do not support suction behavior and are considered remnants of LCs3 that rather serve as a reinforcement of the mouth corners (as seen in *Chlamydoselachus anguineus* in [43]).

## 4. Discussion

Feeding strategies in aquatic vertebrates are quite varied [4,47] (Scheme 1), with many groups using suction-feeding strategies for prey capture, since water is a denser medium than air [48]. Our results show that, in sharks, the number and size of LCs and LC combinations are very diverse and reflect the corresponding feeding strategies, ranging from pure ram feeding to all sorts of combinations and pure suction. They display a functional signal that correlates with their ecological niche and should not solely be used for phylogenetic classification [49].

There are several postulations concerning the feeding process that can be derived from the number, position, orientation, and size of the LCs in relation to feeding strategies in sharks. Species without LCs or with only one small LC1 in a position along the posterior half of the jaws (LC1 Types D and H and Subtypes iii and iv) use pure ram feeding or simple biting as the main feeding strategies (compare with [50,51,52]), whereas the opposite assembly of LCs (high number, strong shape, and anterior position) is related to the use of pure suction (compare with [8,16,28]).

To predict the effectiveness of suction performance during prey capture, we propose employing the following aspects in the following order:

(1) **Number of LC pairs**: 0–1 = no suction; 2–3 = suction of different intensity used in combination with ram or biting behavior; and 4–5 = strong suction resulting in suction as the predominant or even exclusive feeding mechanism.
(2) **Position of the LCs**: The further anterior the LCs 1 and 3 are located, the larger the volume of the mouth cavity. The strongest suction is developed when the LCs are in their extended position and the mouth opening is at its peak [8,53]. LCs 2, 2.1, and 3.1 can be aligned with LCs 1 or 3, or parallel to them, which again influences the mouth volume when the mouth is opened. Aligned LCs provide a volume enhancement, while parallel LCs reinforce the existing LCs and thus strengthen the lateral mouth gape walls, enabling them to bear a stronger suction and possibly can somewhat move along the other LCs.
(3) **Orientation of the LCs:** The more horizontal the LCs are oriented in their resting position, e.g., when the mouth is closed, the larger their movement when the mouth is opened, since they then move antero-labially [28]. This results in a greater enlargement of the mouth cavity and therefore the volume, increasing the suction force. An LC that protrudes labially provides additional support for a larger mouth volume and therefore a stronger suction.
(4) **Size of the LCs:** The larger a LC is, the more force it can endure, which influences the possible suction strength.

### 4.1. Number of Labial Cartilages

Small numbers (1–2 pairs) or a complete lack of LCs as well as amplifications to four or five pairs can be considered divergences from the mean number of three pairs occurring in most species that possess LCs. Zero to one LCs are found in some of the most prominent ram-feeding apex predators. In the great white shark (*Carcharodon carcharias*) or the tiger shark (*Galeocerdo cuvier*), no LCs are developed at all. Other known ram-feeding species, such as the blue shark (*Prionace glauca*) or the bonnethead shark (*Sphyrna tiburo*), possess only one pair of very small LCs, which we consider to represent very reduced remnants having no function for creating suction. This led us to the conclusion that 0–1 LC pairs are a good predictor of pure ram feeding or biting behavior.

Two to three LCs are found in species that use a combination of suction and biting and/or ram feeding. Some of these sharks are generalists (e.g., *Chiloscyllium plagiosum* in [39] and *Galeus melastomus* in [54]), while others display a very distinct hunting behavior (e.g., *Heterodontus francisci* in [18], *Rhincodon typus* in [55], and *Isistius brasiliensis* in [56]).

Four to five LCs are found typically in ambush predators, such as the Japanese wobbegong shark (*Orectolobus japonicas*) or the nurse shark (*Ginglymostoma cirratum*), which predominantly employ effective suction to capture prey [16,28]. This led us to the conclusion that a high number of LCs reliably indicates a suction feeding behavior with a strong suction performance.

### 4.2. Shapes of Labial Cartilages

The general shape of single LCs provides an insight into the movement capabilities of the whole LC apparatus. Small, roundish LCs are likely to serve as a kind of pivoting point, allowing a stronger rotation of the LC apparatus, while broad, elongated LCs add to the enlargement of the mouth cavity volume and LCs with a forking in their posterior end are assumed to provide a stronger connection to the next aligned LC, forming a joint with higher stability and therefore capable of enduring a stronger suction. The longer the LC, the more it adds to the total length of the complete LC arch and, therefore, allows for a larger mouth volume extension when the mouth is opened, which induces suction. Short, thin LCs are often located midway along the jaws or along the posterior half; we assume that they strengthen the mouth corners to prevent the prey from escaping [24], rather than being capable of creating a significant suction.

### 4.3. Labial Cartilage Mobility and Adaptations for Special Feeding Strategies

The range of mobility seemingly increases if three or more LCs are developed. This relates to the fact that at least one of those LCs is not connected tightly to the jaw by ligaments and, therefore, allows a greater moveability. We postulate that the more freely moveable the LCs are, the greater is their influence on the feeding strategy (additionally to the above-stated number, position, orientation, and size). We assume that either the LC2 can partially slide over LC1 and therefore can extend the LC arch, similar to the movement actions of the premaxilla and maxilla in suction-feeding teleosts [57], or the LC2 reinforces the LC1, making this arrangement more robust and capable of enduring higher pressures in species in which the LC2 is located dorsally to LC1.

In the cookie-cutter shark (*Isistius brasiliensis*), the small LC1 can be considered to work as a rotation point, which extends the anatomically possible movement of LC2 labially, forming an entirely ovoid mouth opening that attaches perfectly to a smooth surface leaving no gaps. Also, Jones [58] described its basihyoid cartilage to be able to change its position so that it completely separates the mouth cavity from the pharynx. These modifications allow the cookie-cutter shark to generate a longer-lasting suction, comparable to that of a suction cup. This matches the current state of knowledge about the feeding strategy of the cookie-cutter shark, as it sucks onto larger prey animals, burying its teeth into the flesh and cutting out pieces of flesh by rotating around its own body axis, therefore combining suction and biting in its hunting strategy [56,58,59,60].

According to the LC arrangement, the kitefin shark (*Dalatias licha*) and probably also the pygmy shark (*Euprotomicrus bispinatus*), of which we cannot be sure due to the bad quality of the scan, might use a similar feeding strategy to that of the cookie-cutter shark or at least are capable of producing a suction of similar strength to that of the cookie-cutter shark.

Members of the Heterodontidae have developed another hunting strategy that is also reflected in their LC arrangement. Horn sharks mainly feed on benthic, sessile, or slow-moving prey. Usually, they make contact with the prey item with their snout first and then initialize a short suction strike, whose strength is comparable to the suction used by nurse sharks [4,16,18,47,53]. Their LCs differ from those of all other sharks since they are oriented in a unique way: from a lateral perspective, they resemble an arrow with the tip pointing slightly anteriorly (whereas in all other sharks it is pointing posteriorly); and from a frontal perspective, it can be seen that the LCs stick out labially, even during the resting position (mouth closed). The surrounding labial tissue is comparatively massive in horn sharks and allows a very rapid anterior movement of the LCs, which results in the observed short, but strong suction burst, enabling a precise hunting strike on a short distance. Also, the mouth opening is very small, which additionally intensifies the suction effect. The LCs move anteriorly when the jaw extension reaches its maximum [18], providing contact with the surface or the prey and therefore also preventing the prey from escaping, as was previously suggested by Huber et al. [61].

Many benthic living shark species, like angel sharks (Squatiniformes) or some members of the carpet sharks (Orectolobiformes), specialize in ambush predation. They stay motionless on the ground until a potential prey comes within a certain range of their mouth and then rapidly expand their mouth cavity to create a strong suction that pulls the prey deep into the enlarged mouth cavity [27,40,44,62]. This hunting behavior is reflected in their LC apparatus too: All ambush predators possess at least three, sometimes even four or five pairs of LCs, which are located at the anterior end of the jaws. There are three possibilities in LC arrangement: (1) three pairs of massive LCs (e.g., *Squatina africana*), (2) four pairs of medium-sized LCs with an onset at the anterior tip of the jaws (e.g., *Chiloscyllium punctatum*), and (3) five pairs of massive LCs, with LC2.1 reinforcing LC1 and LC2 displaying a distinct posterior forking (e.g., *Orectolobus japonicas*). In the case of arrangements (2) and (3), the middle joint is formed by LC2 and the smaller, roundish LC3.1.

The whitetail dogfish (*Scymnodalatias albicauda*) is one of the least known species. It also displays a unique set of LCs that does not match any other combination, which makes suggesting a feeding strategy hard, but according to the scarce knowledge of its behavior and its overall body shape, we suggest that it is only capable of producing a minor suction force or no suction at all.

### 4.4. Labial Cartilage Combinations

The possible LC combinations and their relation to the suction capability of the corresponding shark species are depicted in Table 2 (For a list of all the feeding mechanisms of the discussed species, see Supplementary Table S2).

## 5. Conclusions

Based on the knowledge of the feeding strategies in some species and the collected data on labial cartilages (LCs) of over 100 shark species, we were able to relate specific LC apparatus constellations to certain feeding strategies and consequently also to their probable hunting behaviors. The detailed account of the skeletal structures provided in this study allows some general and specific conclusions: (1) Sharks practicing ram feeding either lack LCs completely or only possess remnants of LCs, either of only LC1 or of LC1 and LC3, which then are generally located in the middle or in the posterior half of the jaws. Those sharks mainly occupy higher trophic levels, including apex predators representing tertiary and quaternary consumers, and the majority lives in pelagic waters, hunting actively. (2) Sharks using suction feeding, conversely, possess higher numbers (up to five pairs) of well-developed LCs that are located in the anterior half of the jaws. Such sharks occupy slightly lower trophic levels, representing mainly secondary consumers (e.g., Orectolobiforms and Heterodontiforms) and are, at least partially, benthic. (3) The whole LC apparatus is necessary to predict suction capabilities and therefore a feeding technique, since not only the number, but also the position, orientation, moveability, and combination of LCs are important contributors. This information and the conclusions derived from our study consequently allow to predict feeding and hunting strategies in rare extant sharks, for which behavioral observations are lacking, or even extinct sharks, for which corresponding behavioral observations are impossible. Such a study was beyond the scope of the present paper, but will be conducted in the near future by the authors. In extant shark species, it might even provide information that is helpful for defining conservation measures to protect sharks and their surrounding ecosystems along with them.

## Figures and Tables

**Table 1:** LC definitions and color-coding from Klimpfinger and Kriwet [26].

Color Code	LC No.	Description
Blue	LC1	Located/onset at the most anterior position along the upper jaw; sometimes connected to the jaw on the anterior end; mostly oriented along the anterior–posterior-axis; variable shapes
Green	LC2	Located between LC1 and LC3 or between LC1 and LC3.1; never connected to the jaw; oriented antero-posteriorly in differing angles to the dorso-ventral axis; shape is elongated; forking on the anterior end in Orectolobidae
Yellow	LC2.1	A segregated part of the forking of LC2 (see [27]); located dorsally to that of the LC1; never connected to the jaw; orientated antero-posteriorly; slender cartilage only present in Orectolobidae
Orange	LC3.1	A segregated part of the LC3 (see [27]); located in the more posterior position along the lower jaw between LC2 and LC3; oriented antero-posteriorly; mostly short and stout
Red	LC3	Located at the most anterior position along the lower jaw; often connected to the jaw on the anterior ventral end; oriented antero-posteriorly in differing angles to the dorso-ventral axis; variable shape

**Table 2:** Combinations of LCs, the corresponding suction capabilities, and the suspected feeding strategy it leads to (marked by an arrow).

LC1	LC2	LC2.1	LC3.1	LC3	Suction Capabilities	Example Species from the Dataset
1LC only (no matter which)					no suction → biting or ram	Blue shark (*Prionace glauca*)
D/H/iii	-	-	-	G		Lemon shark (*Negaprion brevirostris*)
V	-	-	-	ii	no suction → extreme jaw protrusion	Goblin shark (*Mitsukurina owstonii*)
H	-	-	-	E	minor to no suction → biting	Small-tooth sand tiger (*Odontaspis ferox*)
H	A	-	B	iii	minor to no suction (uncertain)	Whitetail dogfish (*Scymnodalatias albicauda*)
A/E/F	(i)	-	-	B	minor suction → generalist, mainly biting	Blacktip sawtail catshark (*Galeus sauteri*)
A/E/G	-	-	-	E/F		Smooth-hound (*Mustelus mustelus*)
G	-	-	-	C		Blackbelly lanternshark (*Etmopterus lucifer*)
iv	i	-	-	i		Frilled shark (*Chlamydoselachus anguineus*)
H	A	-	-	E		Barbeled houndshark (*Leptocharias smithii*)
E	ii	-	-	F	medium to minor suction → probably scavenger	Bramble shark (*Echinorhinus brucus*)
E	A	-	-	A		Mosaic gulper shark (*Centrophorus tesselatus*)
B/C/G/vi	C	-	(A)	E/F	medium suction + biting or ram → more effective when close to the ground or the surface	Whale shark (*Rhincodon typus*)
A/E/G	A	-	-	A/D/F	medium suction → generalist, mainly benthic prey	Knifetooth dogfish (*Scymnodon ringens*)
A/B/G	A/ii/iii	-	-	C		Angular roughshark (*Oxynotus centrina*)
B	-	-	-	B		Redspotted catshark (*Schroederichthys chilensis*)
A/ii	A/B	-	-	A	strong to medium suction → capable of longer-lasting suction (comparable to a suction cup); used for ectoparasitism	Cookie-cutter shark (*Isistius brasiliensis*)
vii	-	-	-	F	strong, short suction → mainly sessile prey	Horn shark (*Heterodontus francisci*)
E/F	C	A	A	B	strong suction → benthic ambush predator	Japanese wobbegong (*Orectolobus japonicus*)
A/E/F/i	B	-	(C)	E	strong suction → benthic ambush predator leaping from the bottom for strikes	Angelshark (*Squatina squatina*)
B/F/i	A	-	B	C/F	strong suction → benthic generalist	Nurse shark (*Ginglymostoma cirratum*)

## Data Availability

The data presented in this study are openly available in https://sharksrays.org/; https://figshare.com/.

## Supplementary Materials

The following supporting information can be downloaded at: https://www.mdpi.com/article/10.3390/biology12121486/s1, Table S1: species overview; Table S2: feeding mechanisms. References [17,18,27,28,37,39,41,42,43,44,45,46,50,55,56,58,59,61,62,63,64,65,66,67] are cited in the supplementary materials.
