# Supplementary material for: Morphological Variability and Function of Labial Cartilages in Sharks (Chondrichthyes, Elasmobranchii)
Source: Biology (Basel). 2023 Dec 3;12(12):1486. doi: 10.3390/biology12121486 (PMC10741050; doi:10.3390/biology12121486)
Supplement: Supplementary file 1 [file biology-12-01486-s001.zip › Supplementary Table1_SpeciesOverview_120species.pdf]

Superorder	Order	Family	Gattung	Art	Total Number of LC-Pairs	LC1	LC2	LC2.1	LC3.1	LC3
SQUALOMORPHII	Hexanchiformes	Chlamydoselachidae	Chlamydoselachus	C. anguineus	3	1	1	0	0	1
		Hexanchidae	Heptranchias	H. perlo	0	0	0	0	0	0
			Hexanchus	H. nakamurai	1	1	0	0	0	0
	Squaliformes	Centrophoridae	Centrophorus	C. seychellorum	3	1	1	0	0	1
				C. uyato	3	1	1	0	0	1
				C. tessellatus	3	1	1	0	0	1
			Deania	D. calcea	3	1	1	0	0	1
		Dalatiidae	Mollisquama	M. parini	3	1	1	0	0	1
			Dalatias	D. licha	3	1	1	0	0	1
			Isistius	I. brasiliensis	3	1	1	0	0	1
			Euprotomicrus	E. bispinatus	1	1	0	0	0	0
		Etmopteridae	Etmopterus	E. lucifer	2	1	0	0	0	1
				E. sheikoi	2	1	0	0	0	1
				E. splenndidus	2	1	0	0	0	1
				E. spinax	0	0	0	0	0	0
			Trigonognathus	T. kabeyai	0	0	0	0	0	0
		Oxynotidae	Oxynotus	O. centrina	3	1	1	0	0	1
		Somniosidae	Centroscymnus	C. crepidater	3	1	1	0	0	1
				C. owstonii	3	1	1	0	0	1
			Scymnodalatias	S. albicauda	4	1	1	1	0	1
			Scymnodon	S. ringens	3	1	1	0	0	1
			Somniosus	S. microcephalus [White 1895]	0	0	0	0	0	0
			Zameus	Z. squamosum	3	1	1	0	0	1
		Squalidae	Squalus	S. acanthias	3	1	1	0	0	1
				S. suckleyi	3	1	1	0	0	1
				S. cubensis	3	1	1	0	0	1
				S. megalops	3	1	1	0	0	1
				S. mitsukurii	3	1	?	0	0	1
				S. brevirostris	3	1	1	0	0	1
	Echinorhiniformes	Echinorhinidae	Echinorhinus	E. brucus = E. spinosus	3	1	1	0	0	1
	Squatiniformes	Squatinidae	Squatina	S. squatina	3	1	1	0	0	1
				S. africana	4	1	1	0	1	1
				S. japonica	3	1	1	0	0	1
				S. nebulosa	3	1	1	0	0	1
	Pristiophoriformes	Pristiophoridae	Pristiophorus	P. japonicus	0	0	0	0	0	0
				P. nudipinnis	1	1	0	0	0	0
	Heterodontiformes	Heterodontidae	Heterodontus	H. japonicas	2	1	0	0	0	1
				H. francisci	2	1	0	0	0	1
				H. portusjacksoni [Summers 2004]	2	1	0	0	0	1
		Brachaeluridae	Brachaelurus	B. waddi	3	1	1	0	0	1
		Ginglymostomatidae	Ginglymostoma	G. cirratum	3	1	1	0	0	1
			Nebrius	N. ferrugineus	3	1	1	0	0	1
			Chiloscyllium	C. hasselti	3	1	1	0	0	1
				C. arabicum	3	1	1	0	0	1
				C. indicum	3	1	1	0	0	1

GALEOMORPHII	Orectolobiformes	Hemiscylliidae	[illegible]	C. punctatum	4	1	1	0	1	1
				C. griseum	4	1	1	0	1	1
				C. plagiosum	3	1	1	0	0	1
			Hemiscyllium	H. trispeculare	4	1	1	0	1	1
				H. strahani	4	1	1	0	1	1
				H. ocellatum	4	1	1	1	0	1
		Orectolobidae	Eucrossorhinus	E. dasypogon	5	1	1	1	1	1
			Orectolobus	O. japonicus	5	1	1	1	1	1
				O. maculatus	5	1	1	1	1	1
		Parascylliidae	Parascyllium	P. collare	4	1	1	1	0	1
		Rhincodontidae	Rhincodon	R. typus [Denison 1937]	3	1	1	0	0	1
		Stegostomatidae	Stegostoma	S. fasciatum	4	1	1	0	1	1
	Lamniformes	Alopiidae	Alopias	A. vulpinus	1	1	0	0	0	0
				A. superciliosus	0	0	0	0	0	0
		Cetorhinidae	Cetorhinus	C. maximus	0	0	0	0	0	0
		Lamnidae	Carcharodon	C. carcharias [Shimada 2009]	0	0	0	0	0	0
			Isurus	I. oxyrinchus [Shimada 2009]	0	0	0	0	0	0
				I. paucus [Shimada 2009]	0	0	0	0	0	0
			Lamna	L. ditropis [Shimada 2009]	0	0	0	0	0	0
				L. nasus	0	0	0	0	0	0
		Megachasmidae	Megachasma	M. pelagios [Seigel 1985 / Shimada 2009]	0	0	0	0	0	0
		Mitsukurinidae	Mitsukurina	M. owstoni	2	1	0	0	0	1
		Odontaspididae	Carcharias	C. taurus	0	0	0	0	0	0
			Odontaspis	O. ferox [Shimada 2009]	2	1	0	0	0	1
		Pseudocarchariidae	Pseudocarcharias	P. kamoharai	0	0	0	0	0	0
		?	Galeocerdo	G. cuvier [pers. obs. Simon de Marchi]	0	0	0	0	0	0
		Carcharhinidae	Scoliodon	S. laticaudus	1	1	0	0	0	0
				S. macrorhynchos	0	0	0	0	0	0
			Carcharhinus	C. falciformis	2	1	0	0	0	1
				C. macloti	1	1	0	0	0	0
				C. amboinensis	0	0	0	0	0	0
				C. hemiodon	0	0	0	0	0	0
				C. leucas	0	0	0	0	0	0
				C. melanopterus	0	0	0	0	0	0
				C. galapagensis	2	?	0	0	0	?
				C. plumbeus	0	0	0	0	0	0
			Prionace	P. glauca	1	1	0	0	0	0
			Negaprion	N. brevirostris	2	1	0	0	0	1
			Rhizoprionodon	R. terraenovae	0	0	0	0	0	0
			Isogomphodon	I. oxyrhynchus	0	0	0	0	0	0
			Triaenodon	T. obesus	0	0	0	0	0	0
		Hemigaleidae	Chaenogaleus	C. macrostoma	2	1	0	0	0	1
			Hemigaleus	H. microstoma	1	1	0	0	0	0
			Hemipristis	H. elongatus	2	1	0	0	0	1
		Leptochariidae	Leptocharias	L. smithii	2	1	0	0	0	1
		Proscylliidae	Eridacnis	E. radcliffei	0-1	1	0	0	0	0
			Apristurus	A. laurussonii	2	1	0	0	0	1
				A. macrostomus	2	1	0	0	0	1

	Carcharhiniformes	Scyliorhinidae	Atelomycterus	A. marmoratus	2	1	0	0	0	1
				A. macleayi	2	1	0	0	0	1
			Bythaelurus	B. canescens	2	1	0	0	0	1
			Cephaloscyllium	C. ventriosum	0	0	0	0	0	0
			Galeus	G. melastomus	2	1	0	0	0	1
				G. sauteri	3	1	?	0	0	1
			Halaelurus	H. boesemani	0	0	0	0	0	0
				H. buergeri	0	0	0	0	0	0
			Poroderma	P. africanum	1	1	0	0	0	0
			Schroederichthys	S. chilensis	2	1	?	0	0	1
			Scyliorhinus	S. boa	1	1	0	0	0	0
				S. canicula	1	1	0	0	0	0
				S. stellaris	1	1	0	0	0	0
				S. meadi	1	1	0	0	0	0
		Sphyrnidae	Eusphyra	E. blochii	0	0	0	0	0	0
			Sphyrna	S. zygaena	0	0	0	0	0	0
				S. lewini	1	1	0	0	0	0
				S. corona	0	0	0	0	0	0
				S. tiburo	0	0	0	0	0	0
				S. tudes	0	0	0	0	0	0
				S. media	0	0	0	0	0	0
		Triakidae	Galeorhinus	G. galeus	2	1	0	0	0	1
			Mustelus	M. mustelus	2	1	0	0	0	1
				M. higmani	1	1	0	0	0	0
				M. asterias	2	1	0	0	0	1
				M. manazo	2	1	?	0	0	1
			Triakis	T. semifasciata	2	1	0	0	0	1

- 34 x 0 LC
- 16 x 1 LC
- 28 x 2 LC
- 30 x 3 LC
- 9 x 4 LC
- 3 x 5 LC
