# Supplementary material for: Morphological Variability and Function of Labial Cartilages in Sharks (Chondrichthyes, Elasmobranchii)
Source: Biology (Basel). 2023 Dec 3;12(12):1486. doi: 10.3390/biology12121486 (PMC10741050; doi:10.3390/biology12121486)
Supplement: Supplementary file 1 [file biology-12-01486-s001.zip › Supplementary Table2_FeedingMechanisms120species.pdf]

Family	Gattung	Art	Total Number of LC-Pairs	Feeding Method	Reference (R) or Deduced from Data (D)
Chlamydoselachidae	Chlamydoselachus	C. anguineus	3	ram + biting	R: Wilga 2002
Hexanchidae	Heptranchias	H. perlo	0	Biting	R: Kryukova & Kuznetsov 2020
	Hexanchus	H. nakamurai	1	biting + suction	D
Centrophoridae	Centrophorus	C. seychellorum	3	biting + suction	D
		C. uyato	3	biting + suction	D
		C. tessellatus	3	biting + suction	D
	Deania	D. calcea	3	biting + suction	D
Dalatiidae	Mollisquama	M. parini	3	biting + suction	R: Denton et al. 2018
	Dalatias	D. licha	3	biting + suction	D
	Isistius	I. brasiliensis	3	biting + suction	R: Jones 1971; Shirai 1992; Figueiredo & Carvahlo 2018; Papastamatiou et al. 2010
	Euprotomicrus	E. bispinatus	1	biting	D
Etmopteridae	Etmopterus	E. lucifer	2	biting	D
		E. sheikoi	2	biting	D
		E. splenndidus	2	biting	D
		E. spinax	0	ram or biting	D
	Trigonognathus	T. kabeyai	0	ram or biting	D
Oxynotidae	Oxynotus	O. centrina	3	biting + suction	R: Capape 2008
Somniosidae	Centroscymnus	C. crepidater	3	biting + suction	D
		C. owstonii	3	biting + suction	D
	Scymnodalatias	S. albicauda	4	?	
	Scymnodon	S. ringens	3	biting + suction	D
	Somniosus	S. microcephalus [White 1895]	3	suction + biting	R: Grant et al. 2018
	Zameus	Z. squamosum	3	biting + suction	D
Squalidae	Squalus	S. acanthias	3	suction + biting (+ram)	R: Wilga 2001 & 2007
		S. suckleyi	3	biting + suction	D
		S. cubensis	3	biting + suction	D
		S. megalops	3	biting + suction	D
		S. mitsukurii	3	biting + suction	D
		S. brevirostris	3	biting + suction	D
Echinorhinidae	Echinorhinus	E. brucus = E. spinosus	3	ram + suction	D
Squatinidae	Squatina	S. squatina	3	suction	D
		S. africana	4	suction	D
		S. japonica	3	suction	D
		S. nebulosa	3	suction	D

Pristiophoridae	Pristiophorus	P. japonicus	0	ram or biting	D
		P. nudipinnis	1	ram or biting	D
Heterodontidae	Heterodontus	H. japonicas	2	suction + biting	D
		H. francisci	2	suction + biting	R: Edmonds et al. 2001; Huber et al. 2005
		H. portusjacksoni [Summers 2004]	2	suction + biting	D
Brachaeluridae	Brachaelurus	B. waddi	3	biting + suction	D
Ginglymostomatidae	Ginglymostoma	G. cirratum	3	suction	R: Motta & Wilga 1999; Motta et al. 2002 & 2008; Wilga 2007; Gardiner et al. 2017
	Nebrius	N. ferrugineus	3	suction	D
Hemiscylliidae	Chiloscyllium	C. hasselti	3	biting + suction	D
		C. arabicum	3	biting + suction	D
		C. indicum	3	biting + suction	D
		C. punctatum	4	suction	D
		C. griseum	4	suction	D
		C. plagiosum	3	suction	R: Nauwelaerts 2007; Wilga 2007; Scott et al. 2019
	Hemiscyllium	H. trispeculare	4	suction	D
		H. strahani	4	suction	D
		H. ocellatum	4	suction + biting	R: Wu 1994
Orectolobidae	Eucrossorhinus	E. dasypogon	5	suction	D
	Orectolobus	O. japonicus	5	suction	D
		O. maculatus	5	suction	R: Wu 1994
Parascylliidae	Parascyllium	P. collare	4	biting + suction	D
Rhincodontidae	Rhincodon	R. typus [Denison 1937]	3	ram-filterfeeding or suction	R: Cade et al. 2020; Motta et al. 2010
Stegostomatidae	Stegostoma	S. fasciatum	4	suction + biting	D
Alopiidae	Alopias	A. vulpinus	1	ram + biting	D
		A. superciliosus	0	ram + biting	D
Cetorhinidae	Cetorhinus	C. maximus	0	ram	D
Lamnidae	Carcharodon	C. carcharias [Shimada 2009]	0	ram + biting	R: Tricas 1985
	Isurus	I. oxyrinchus [Shimada 2009]	0	ram (biting)	D
		I. paucus [Shimada 2009]	0	ram	D
	Lamna	L. ditropis [Shimada 2009]	0	ram (biting)	D
		L. nasus	0	ram	D
Megachasmidae	Megachasma	M. pelagios [Shimada 2009]	0	ram-filterfeeding	R: Tomita et al. 2011
Mitsukurinidae	Mitsukurina	M. owstoni	2	ram + biting	D
Odontaspididae	Carcharias	C. taurus	0	biting	D
	Odontaspis	O. ferox [Shimada 2009]	2	biting	R: Wilga 2007
Pseudocarchariidae	Pseudocarcharias	P. kamoharai	0	ram	D
?	Galeocerdo	G. cuvier [pers. obs. de Marchi]	0	ram + biting	D

Carcharhinidae	Scoliodon	S. laticaudus	1	ram or biting	D
		S. macrorhynchos	0	ram	D
	Carcharhinus	C. falciformis	2	ram (biting)	D
		C. macloti	1	biting	D
		C. amboinensis	0	ram	D
		C. hemiodon	0	ram	D
		C. leucas	0	ram	D
		C. melanopterus	0	ram	R: Gardiner et al 2017
		C. galapagensis	2	ram + suction	D
		C. plumbeus	0	biting	R: Ramsay 2012
	Prionace	P. glauca	1	ram (biting)	D
	Negaprion	N. brevirostris	2	ram	R: Motta et al. 1995
	Rhizoprionodon	R. terraenovae	0	ram	D
	Isogomphodon	I. oxyrhynchus	0	ram	D
	Triaenodon	T. obesus	0	ram (biting)	D
Hemigaleidae	Chaenogaleus	C. macrostoma	2	ram + biting	D
	Hemigaleus	H. microstoma	1	biting	D
	Hemipristis	H. elongatus	2	biting	D
Leptochariidae	Leptocharias	L. smithii	2	biting (suction)	D
Proscylliidae	Eridacnis	E. radcliffei	0-1	biting	D
Scyliorhinidae	Apristurus	A. laurussonii	2	biting (suction)	D
		A. macrostomus	2	biting (suction)	D
	Atelomycterus	A. marmoratus	2	biting (suction)	D
		A. macleayi	2	biting (suction)	D
	Bythaelurus	B. canescens	2	biting	D
	Cephaloscyllium	C. ventriosum	0	ram	D
	Galeus	G. melastomus	2	biting (suction)	D
		G. sauteri	3	biting (suction)	D
	Halaelurus	H. boesemani	0	biting or ram	D
		H. buergeri	0	biting or ram	D
	Poroderma	P. africanum	1	biting	D
	Schroederichthys	S. chilensis	2	suction + biting	D
	Scyliorhinus	S. boa	1	biting	D
		S. canicula	1	biting	D
		S. stellaris	1	biting	D
		S. meadi	1	biting	D
	Eusphyra	E. blochii	0	ram	D

Sphyrnidae	Sphyrna	S. zygaena	0	ram (biting)	D
		S. lewini	1	ram (biting)	D
		S. corona	0	ram	D
		S. tiburo	0	ram (biting)	R: Gardiner et al. 2017; Wilga 2001
		S. tudes	0	ram	D
		S. media	0	ram	D
Triakidae	Galeorhinus	G. galeus	2	biting (suction)	D
	Mustelus	M. mustelus	2	biting (suction)	D
		M. higmani	1	biting (ram)	D
		M. asterias	2	biting (suction)	D
		M. manazo	2	biting (suction)	D
	Triakis	T. semifasciata	2	suction + biting	D
